# Past and Future Explanations for Depersonalization and Derealization Disorder: A Role for Predictive Coding

**DOI:** 10.3389/fnhum.2022.744487

**Published:** 2022-03-07

**Authors:** Andrew Gatus, Graham Jamieson, Bruce Stevenson

**Affiliations:** Faculty of Medicine and Health, School of Psychology, University of New England, Armidale, NSW, Australia

**Keywords:** depersonalization, derealization, interoception, predictive coding, heartbeat evoked potentials, virtual reality

## Abstract

Depersonalization (DP) and derealization (DR) refer to states of dissociation in which one feels a sense of alienation in relation to one’s self and environment, respectively. Whilst transient episodes often diminish without treatment, chronic experiences of DP and DR may last for years, with common treatments lacking a strong evidence base for their efficacy. We propose a theoretical explanation of DP and DR based on interoceptive predictive coding, and discuss how transient experiences of DP and DR may be induced in the non-clinical population using virtual reality. Further, we review the use of heartbeat evoked potentials in detecting the neural correlates of DP and DR allowing for an objective measure of these experiences in the non-clinical population. Finally, we discuss how the induction and detection of transient experiences of DP and DR in the non-clinical population could shed light on how the brain constructs one’s sense of self and reality.

## Introduction

Depersonalization (DP) and derealization (DR) are described as states of dissociation in which one feels a sense of alienation in relation to one’s self and environment, respectively (American Psychiatric Association [APA], 2013; Kolev et al., 2014). These experiences are often accompanied by a sense of disembodiment (desomatisation) and blunting of emotional responses (Somer et al., 2013). These experiences can be encapsulated by the subjective reports of those with DP and DR, where such descriptions include as if “being in a bubble” or “separated from the world by an invisible barrier such as a pane of glass, a fog, or a veil” (Sierra and David, 2011; Sierra, 2012). Further, the quality of one’s thoughts, body, and surroundings are reported as seeming “unreal, automated, and remote,” with a sense of detachment and estrangement from one’s thinking, body and world (World Health Organization [WHO], 1992; Sierra and David, 2011). DP and DR can occur individually or together as transient episodes in the general population, as a chronic condition in its own right (depersonalization/derealization disorder; DDD; American Psychiatric Association [APA], 2013), as a comorbid condition alongside other mental health disorders (e.g., panic disorder, generalized anxiety disorder, post-traumatic stress disorder; Medford, 2012), and following drug use, stress, and medical conditions (i.e., sickness and brain damage; Simeon et al., 2003; Kolev et al., 2014). Whilst transient episodes, such as those lasting minutes to weeks, often diminish without treatment, chronic experiences of DP and DR may last for years, with common treatments such as pharmacological intervention, psychological intervention, and transcranial magnetic stimulation (rTMS) failing to show reliable symptom reduction (Somer et al., 2013; Michal et al., 2016).

As evidence of the efficacy of current treatment remains limited (Medford et al., 2005; Somer et al., 2013; Hunter et al., 2017), we suggest treatment protocols could be improved by:

1. Developing a deeper understanding of the causal pathways underlying DP and DR experiences.
2. Examining DP and DR in isolation as experiences separate from other clinical conditions.
3. Developing reliable methods with which to induce experiences of DP and DR for investigation in psychologically normal individuals.
4. Further developing methods that reliably capture the objective correlates of DP and DR within the human body in conjunction with self-report.

In order to address the above, this review presents a theoretical model of DP and DR capable of explaining these experiences in isolation; reviews the use of the heartbeat evoked potential (HEP) as a measure of the biological correlates of DP and DR; and proposes an experimental design which induces and captures the neural correlates of DP and DR using virtual reality, which we believe will help to contribute to the development of an effective treatment for DP and DR.

Whilst earlier accounts of DP and DR suggest alterations of autonomic responses (as commonly measured by skin conductance responses; SCRs) and altered activity in neural regions believed to be responsible for the generation of emotional responses (such as the amygdala and insula), leads to a lack of emotional coloring of one’s own experiences as found in DP and DR (Sierra and Berrios, 1998; Phillips et al., 2001; Phillips and Sierra, 2003; Lemche et al., 2008), our model will propose a multi-sensory, interoceptive predictive coding account of DP and DR in line with more recent theories (Friston et al., 2009, 2010; Seth et al., 2011; Clark, 2013). In particular, we will focus on the role of interoceptive predictive processing in the generation of emotions and the maintenance of one’s felt sense of connection to the physical body and external environment. Further, we will discuss how the precision of interoceptive predictions relative to other sensory modalities is implicated in the generation of DP and DR states. We will then go on to discuss the need for developing more reliable methods of inducing DP and DR in isolation, which could be achieved through manipulating the relative prediction precision of sensory modalities that contribute to one’s overall perception in a virtual reality (VR) environment. Finally, we will discuss how the simultaneous induction of DP and DR in a VR environment whilst measuring neural correlates of interoceptive predictive processing could lead to an objective identification of DP and DR states, further clarifying the differing neural responses between these experiences.

## Interoception and Depersonalization/Derealization

It has been frequently proposed that feeling states result from predictions for the interoceptive state of the body (James, 1890; Schachter and Singer, 1962; Seth et al., 2011). If true, then interoceptive processing may be implicated in the decreased emotional responsiveness and loss of felt connection to one’s physical body and environment experienced by those with DDD (Michal et al., 2014; Medford et al., 2016; Seth and Friston, 2016). Whilst definitions of interoception vary between authors (see Ceunen et al., 2016, for a review), it more frequently refers to the perception of the physiological state of the body, such as one’s heart-rate, hunger, visceral and muscular sensations, and other homeostatic functions (Critchley et al., 2004; Seth et al., 2011; Garfinkel et al., 2015). Interoception can be distinguished from exteroception, which refers to perception of the environment external to one’s body via the five classical sensory modalities, and proprioception and kinesthesia, which reflect the position and movement of the body in space, respectively (Seth et al., 2011). We will thus refer to interoception, proprioception (including kinesthesia) and exteroception as separate, yet related aspects of perception for the remainder of this review.

Physical markers of interoception include measurements of heart rate (Schandry, 1981; Katkin et al., 1983; Brener and Kluvitse, 1988) skin conductance (Sierra et al., 2002a; Krautwurst et al., 2016), functional magnetic resonance imaging (fMRI; Critchley et al., 2004), and electroencephalography (EEG; Herbert et al., 2007). Often these physical markers are paired with self-report data in order to obtain a measurement of interoception. For instance, electrocardiogram (ECG) data combined with self-reported perception of one’s heart rate is commonly used to determine sensitivity to and accuracy of interoceptive signals (Kleckner et al., 2015). Similarly, concurrent measurement of non-specific skin conductance responses and self-monitoring of physiological states are used to determine interoceptive sensitivity (Krautwurst et al., 2014). More recent studies have utilized EEG and fMRI during self-monitoring of interoceptive signals (e.g., heart rate) to identify electrical neural activity in common brain regions, which have been associated with both interoceptive processing and the experience of emotion. These brain regions include the anterior insula cortex (AIC) and the anterior cingulate cortex (ACC; Schulz, 2016; Duquette, 2017; Stern et al., 2017; Wang et al., 2019). In particular, the AIC is believed to be the cortical center for information processing of the viscera and subsequent interoceptive states, where such processes have less specifically been linked to the generation of emotional states themselves (Gu et al., 2013). For instance, AIC activation has been correlated with recall of autobiographical emotional experiences (such as sadness or anger), sexual arousal, anticipatory anxiety and pain, and panic, where AIC neurons have been found to innervate viscera directly and indirectly (e.g., smooth muscle; Craig, 2002; Wager, 2002; Gu et al., 2013; Uddin et al., 2017). Additionally, the ACC is believed to mediate the allocation of attentional resources to emotionally arousing stimuli (Niedenthal and Kitayama, 1994; Lane et al., 1998; Kanske and Kotz, 2011; Stevens et al., 2011). Due to their functional connectivity, it has been proposed that combined activation of the AIC and ACC works to integrate perception of stimuli and the perceived physiological responses to them, leading to the generation of emotions (Caseras et al., 2013).

The convergence of interoceptive and emotional processing within the AIC and ACC suggests diminished activity in these brain regions may explain symptoms of DP and DR (Medford and Critchley, 2010; Seth et al., 2011). For instance, reduced AIC activity has been associated with attenuation of emotional experience (Medford et al., 2016), and a decline in respiratory and cardiac interoceptive accuracy (i.e., performance on behavioral tests of respiratory and heartbeat detection; Garfinkel et al., 2016; Pollatos et al., 2016). Further, reduced ACC activity has been associated with emotional numbing (i.e., as experienced in PTSD), and increased activation in anxiety, where the presence of anxiety has been associated with more severe DP/DR symptoms (Etkin and Wager, 2007; Stevens et al., 2011; Kolev et al., 2014).

## Interoceptive Predictive Coding

As with all acts of perception, interoceptive processing has traditionally been considered a sensory-driven phenomenon utilizing bottom-up processes (Marshall et al., 2018). However, recent developments in cognitive science have led to a paradigm shift toward a predictive coding computational account of perception and action (Seth and Friston, 2016). While information processing accounts of the brain view mental activity as largely computational in nature (a traditional form involves receiving inputs, processing data, and generating outputs; Piccinini and Bahar, 2013), recent cognitive theories now suggest that information processing involved in perception and belief generation is based on computational models utilizing Bayesian prior probabilities (Clark, 2013). Predictive coding in particular suggests that bottom-up sensory information is compared with top-down predictions (represented as Bayesian probability distributions) regarding the sources of sensory information (Feldman and Friston, 2010; Ainley et al., 2016).

In a predictive coding model, one’s internal generative models attempt to capture the overall statistical structure of sensory data by testing predictions based on these models with sampled sensory data. The system is organized in a hierarchical manner, with descending predictions compared against bottom-up sensory data at multiple levels within the hierarchy (Rauss and Pourtois, 2013). If a mismatch exists between a prediction and sampled sensory data, a prediction error is generated, representing the difference between the predicted and sampled sensory data (Feldman and Friston, 2010; Hohwy, 2012). In order to manage the presence of prediction errors and improve the predictive ability of generative models (to generate accurate percepts), a predictive coding system can either:

1) Suppress or ignore prediction errors, allowing one’s predictions to dominate perception, or,
2) Allow prediction errors to inform and revise the predictions above it within the predictive coding hierarchy, resulting in more accurate predictions during subsequent sampling periods.

More recently, an extended approach to prediction error minimization involving action has been proposed, where behaviors are engaged that alter sensory input to better match predictions, a process known as active inference (Friston et al., 2009, 2010). If prediction error can be managed through suppression/ignoring of errors, plus revision of predictions as previously discussed, active inference allows for an additional means of managing such errors, which can be achieved through changing the sampling of the sensory environment to better match predictions, such as may occur when physiological arousal is increased in response to a prediction of imminent threat (Friston et al., 2011; Adams et al., 2013; Seth and Friston, 2016). This process is believed to operate hierarchically, with different bodily mechanisms responsible for altering sampled sensory data (through action) dependent on the modality of the descending higher-level prediction. For instance, descending interoceptive predictions from higher hierarchical predictions may enslave lower-level autonomic reflex arcs. For proprioceptive predictions, higher level descending predictions may drive the activation of motor reflex arcs (Friston and Kiebel, 2009; Friston, 2012; Seth and Friston, 2016; Stephan et al., 2016). In this way, perception and action become intrinsically linked in exteroceptive, proprioceptive, and interoceptive domains (Adams et al., 2013).

In the absence of active inference, the extent to which prediction errors are suppressed/ignored or predictions revised is dependent upon the variability of predictions, and the consistency of precision (the inverse of variability) of prediction errors (Hohwy, 2012). For instance, in a stable sensory environment, variability (i.e., signal noise) in a sensory signal is expected to be relatively constant, generating consistent prediction errors whose variability may be learned and predicted over time (Hohwy, 2012; Gadsby and Hohwy, 2019; Hsu et al., 2020). In an unstable sensory environment, deviations in the expected variability of prediction errors, which may occur due to random and unexpected signal noise (i.e., misfiring of sensory neurons), leads to prediction errors which carry less informative data in which to adjust predictions. In order to account for noise, a predictive coding system must then carry the ability to register both sensory information *and* the variability of this sensory information as inferred through the magnitude and type of prediction error encountered (i.e., within a specific context), and consistency of prediction error variability (Hohwy, 2012; Ainley et al., 2016; Gadsby and Hohwy, 2019).

The variability of prediction error precision (quantified as the inverse of signal variability) is further weighted against the precision of prediction distributions themselves, which represents the range of potential predictions capable of predicting sensory data, each with varying degrees of probability (Hohwy, 2012). This can be exemplified in situations where one’s predictions are imprecise, as in a relatively unfamiliar environment where one is uncertain of what sensory data to expect. In this scenario, prediction errors are given a greater weighting in determining perception, where these errors are used to update imprecise predictions, allowing for greater predictive capabilities during future sensory sampling periods. When one’s predictions are precise, as in encountering a familiar, well-learned environment, predictions are given greater weighting, resulting in greater suppression of prediction error. For example, one is more likely to rely on spatial predictions of objects’ locations when navigating a dark room following a previous encounter of this environment in the light.

Overall, registered variability in prediction errors is critical for resolving conflicts between predictions and sensory data in acts of perception. As will be discussed, this impacts upon one’s connection to their own body (DP) or external environment (DR).

## Interoceptive Predictive Coding and Clinical Conditions

In addition to predicting mundane interoceptive experiences of our everyday life, including hunger, tiredness, sexual arousal, and so forth, predictive coding accounts of interoception suggest these predictions are responsible for anticipating how the physiological state of the body may change when encountering predicted external (i.e., an attacker) and/or internal (i.e., heart-attack) threats (Paulus and Stein, 2006; Seth et al., 2011; Ainley et al., 2016). This latter process provides insights into the role of interoceptive prediction precision in maintaining certain psychopathologies. For instance, interoceptive predictions of threat engage anticipatory physiological changes (i.e., increased perspiration and heart-rate) in preparation for predicted incoming threat, in line with active inference accounts of interoception (Ainley et al., 2016). It has been proposed that in cases of anxiety disorders, the precision of interoceptive predictions (of threat) may be so great that these predictions are maintained in the absence of actual threat, resulting in sustained, heightened physiological arousal (Paulus and Stein, 2006; Seth et al., 2011; Ainley et al., 2016). Ainley et al. (2016) suggests avoidance of sensory information that would normally disconfirm precise interoceptive predictions of physical threat, and stronger attention toward interoceptive cues (increasing their chance of reaching awareness), may be responsible for such heightened anxiety.

In addition to anxiety, aberrant precision weighting has been implicated in other clinical conditions, including schizophrenia (Synofzik and Voss, 2010), depression (Barrett et al., 2016), and obsessive-compulsive disorder (Levy, 2018). Of particular relevance to experiences of DP and DR are how precision weightings are set not only within the interoceptive sensory modality, but within and between interoception and other sensory modalities. In particular, several recent investigations have found interactions between multi-sensory manipulation tasks and DP and DR. For instance, in experiments utilizing the Rubber Hand Illusion (RHI), a false sense of body ownership occurs during congruent stroking of a rubber hand and real hand whilst visually inspecting stroking of the rubber hand (Botvinick and Cohen, 1998). Whilst sense of agency, body ownership, and bodily location are key constructs often examined during the RHI, successful induction of transient experiences of DP and DR has also previously been reported in those with dissociative subtypes of post-traumatic stress disorder during the RHI illusion, with variability of the illusion itself also greater in these groups (Rabellino et al., 2016, 2018; Rosa et al., 2019). A related investigation by Kanayama et al. (2009) found stronger self-reported illusory effects during the RHI in those with high versus low DP.

Adler et al. (2016) investigated somatosensory evoked potentials (SEPs) generated by a tactile stimulus applied to the cheek in order to investigate the integration of self or other visual information (mirroring) with somatosensory perceptual processing (of the pre-reflective ‘bodily-self’; Krol et al., 2020) in high and low trait depersonalization (TD) groups. Somatosensory stimuli were paired with visual sequences in which the face of oneself or another person was touched by a pencil with the timing of the apparent visual touch and the tactile stimulus synchronized to co-occur. The finding of greatest relevance here was the early P45 component of the SEP. This component was significantly reduced in the high TD group for the self-touch condition but not for the other-touch condition. The P45 closely resembles the P50 event-related potential component found in other sensory modalities, which is highly sensitive to repetition suppression effects (Boutros et al., 2013). From a predictive processing perspective this early component can be interpreted as an initial prediction error signal which reflects the discrepancy between the stimulus and the relevant top-down generative model (Auksztulewicz and Friston, 2016). The visual face-touch sequence provides information predicting the moment of tactile stimulation. From a predictive processing perspective then, for high TD, the visual self-face touch sequence enables the generation of more accurate somatosensory predictions which results in lower prediction errors hence reduced P45 SEP components. This does not occur for other-face touch so it is highly specific to the influence of bodily-self related visual information on the generation of somatosensory predictions and not a more general feature of visual information processing effects in those with high TD.

Similarly, Farmer et al. (2020) used a visual re-mapping of touch paradigm (VRT); a method of manipulating the degree of visual/tactile sensory integration, to investigate the degree of multi-sensory integration in those with high vs. low DP. During this paradigm, tactile stimulation is delivered to each cheek, with the intensity of the stimuli greater on one side. The stronger stimulus has an extinguishing effect on the weaker, reducing the conscious detection of the weaker stimuli. However, this extinction effect is reduced upon viewing congruent visual feedback of the tactile stimuli being delivered to both sides, with this effect (e.g., improved detection of the weaker stimulus upon receiving visual feedback) stronger in those with higher versus lower reported symptoms of DP (Farmer et al., 2020). Finally, conscious pain responders (i.e., those who experience pain in their body upon viewing others in pain) reported more severe symptoms of DP when viewing others in pain compared with non-pain responders, which could suggest a greater influence of interoceptive sensations from exteroceptive processes in these groups (Bowling et al., 2019). These findings provide insights into how disruptions in multi-sensory perception, that may come about during manipulations of exteroceptive, proprioceptive, and interoceptive information can generate DP/DR states in those susceptible to them.

Within the predictive coding framework, these multi-modal effects can be explained by the generation of a sense of selfhood that is dependent upon the integration of exteroceptive, proprioceptive, and interoceptive information at higher (predictive coding) hierarchical levels (James, 1890; Seth, 2013; Suzuki et al., 2013; Marshall et al., 2018; Limanowski and Friston, 2020). More specifically, the over-weighting of non-interoceptive sensory data into the generation of interoceptive predictions in those with DP and DR may be responsible for these experiences, where interoceptive prediction is influenced more so by these other sensory modalities. For instance, in the RHI, a change in one’s sense of self is likely to occur when predictions between modalities are inconsistent with one another, where the modality with the greatest prediction precision dominates perception (Seth, 2013; Suzuki et al., 2013; Zeller et al., 2015). Whilst proprioceptive information normally dictates the perception of one’s bodily position, Zeller et al. (2015, 2016) suggests the precision weighting of proprioceptive prediction errors are down-regulated during the RHI to better manage the conflicting visual/tactile information generated. This results in exteroceptive information (in this instance, vision) influencing the perception of one’s bodily position to a higher degree in those susceptible to the RHI (Suzuki et al., 2013). As will be discussed, an analogous process may be implicated in the generation of DP and DR states, whereby interoceptive predictions are influenced by other sensory modalities akin to how proprioceptive processes are influenced by exteroception in the RHI, leading to the feelings of false body ownership over the rubber hand.

Previous research has highlighted how interoceptive processes specifically are influenced in the RHI, where, for instance, increased activation of the ACC and insula has been observed during simulated threats to the rubber hand in the RHI (Ehrsson et al., 2007). Additionally, Suzuki et al. (2013) demonstrated increased sense of ownership of a rubber hand during a modified version in which individuals received synchronous interoceptive feedback (e.g., cardiac feedback) during the illusion. Finally, reductions in body temperature of one’s real hand during the RHI have been reported, suggesting an influence of this illusion over interoceptive processes (Moseley et al., 2008), however, this finding has been challenged, with some studies unable to reproduce this cooling effect in one’s hand (De Haan et al., 2017). As will be discussed, these interoceptive effects may highlight the role of interoceptive prediction precision in inducing DP/DR states during multi-sensory manipulations such as the RHI.

The relationship between multi-sensory disruptions and psychopathologies is not new. In particular, stronger RHI effects and proprioceptive drift have been reported in those diagnosed with schizophrenia and anorexia nervosa, with reduced effects in autistic groups (see Crespi and Dinsdale, 2019 for a review). From a predictive coding perspective, aberrant multi-sensory integration may depend upon the level of prediction precision between modalities, where stronger precision provides greater weighting to a particular prediction. For instance, previous studies suggest schizophrenic patients possess imprecise predictions about the sensory causes of actions, and therefore rely more strongly on external cues (e.g., exteroception) in perceiving agency (Synofzik et al., 2010; Seth et al., 2011). More so, highly precise interoceptive predictions have been proposed as a potential mechanism governing anorexia nervosa, for example, highly precise interoceptive predictions overcoming interoceptive signals of hunger (Barca and Pezzulo, 2020). Additionally, Van de Cruys et al. (2014) suggests those with autism spectrum disorder possess highly precise predictions across all sensory modalities, resulting in difficulties determining which sources of sensory information should be given weight in determining overall perception of social cues. Thus, when multiple sources of sensory information are normally perceived as arising from a single source (such as visual-auditory information stemming from the ventriloquist effect), these are perceived as separate, unimodal events (Van de Cruys et al., 2014). Finally, Seth et al. (2011) and others (Schäflein et al., 2018; Koreki et al., 2020) suggest imprecise interoceptive predictions may be responsible for experiences of DP and/or DR, as well as certain delusions (e.g., The Cotard Delusion; a set of beliefs that range from having lost one’s organs, blood, or body parts to having lost one’s soul or life), with these delusions emerging in an attempt for the predictive coding system to better account for DP/DR experiences.

Braithwaite et al. (2017) argue imprecise interoceptive predictions may also be responsible for out-body-experiences (OBEs) as well as DP/DR. However, the authors suggest that whilst DDD and OBE overlap in the loss of the subjective sense of self that accompanies these conditions, the latter may involve additional processes that connect the individual to an exocentric point in space, resulting in the false interpretation that this external position is *mine*. Indeed, whilst similarities exist in how these conditions may be subjectively experienced, SCRs were found to be higher in those prone to OBEs during a threat task delivered to an illusory limb, whilst SCRs were suppressed in DDD. This suggests suppression of physiological responses appears to be found within cases of DDD rather than OBEs, providing a physiologically measurable distinction between these two conditions.

If interoceptive processes are being drawn upon during certain multi-sensory manipulations (such as the RHI, as indicated by the aforementioned findings), then instances of DP and DR may emerge in those with imprecise interoceptive predictions due to an increase in the integration of exteroceptive and/or proprioceptive processes required to account for interoceptive prediction error generated during these manipulations. This may suggest why many individuals can experience the illusory effects in the RHI, but only some (i.e., some of those with dissociative subtypes of PTSD, or as suggested, those with imprecise interoceptive predictions) with accompanying increases in DP and DR experiences.

Overall, if the precision of interoceptive predictions is lower *relative* to that of predictions derived from other sensory modalities, then individuals may over-integrate sensory information from other modalities (e.g., draw upon non-interoceptive predictions to perceive interoceptive states) in attempts to reduce interoceptive prediction error, leading to experiences of DP/DR. As will be discussed, this process may emerge when large and variable interoceptive prediction error remains unconstrained, leading to prediction errors that percolate up the predictive coding hierarchy to update predictions. Due to the variability of these errors, predictions would be unable to develop certainty about the types of error to expect, leading to increasing reductions in the overall precision of interoceptive predictions. Exteroceptive and/or proprioceptive predictions may then be sequestered to overcome interoceptive prediction error, as in proprioception drawing on exteroception in the RHI to overcome proprioceptive prediction error.

Drawing upon previously discussed evidence of interoceptive predictive coding and multi-sensory integration, we suggest that DP and DR result from imprecise interoceptive predictions, relative to predictions in other sensory modalities, which are resistant to updating from interoceptive prediction error. Whilst these prediction errors may be resolved in short temporal windows in transient experiences of DP/DR, leading one to increase interoceptive prediction precision by adjusting these predictions based on feedback from interoceptive prediction error, they remain consistently high in chronic experiences of DP/DR. As suggested by Deane (2020), trauma presents a particularly salient example of how this process may occur, where DP and DR are often consequences of such experiences (Kira et al., 2013). For instance, in on-going physical trauma, homeostatic/allostatic states (hence associated interoceptive signals) move significantly out of predicted ranges, generating rapidly fluctuating, large interoceptive prediction errors stemming from severe physical damage, with the individual unable to rapidly change predictive models to accommodate the large prediction errors or engage in active inference in order to reduce prediction error. As such, interoceptive predictions may become increasingly imprecise due to this influx of large and variable prediction errors, where the variability of these errors influences prediction precision by increasing the possible number of predictions available to account for prediction error, but without necessarily increasing their likelihood (Deane, 2020). Extending on this, we further suggest that such experiences could lead to current models of the self that more heavily draw upon exteroceptive and proprioceptive processes, rather than interoceptive processes, during multi-sensory integration within the predictive coding hierarchy. Such processes could lead to many of the subjective experiences described in those who experience DP and DR. For instance, whilst those with DP/DR tend to fixate on their perceived internal (interoceptive) symptoms (Hunter et al., 2014; Michal et al., 2014), attempts to model self-states through exteroceptive and proprioceptive processes, rather than interoceptive processes, may mean this fixated attention is actually directed toward non-interoceptive sources of information, akin to how one generates proprioceptive models using exteroceptive signals to discount proprioceptive prediction errors in the RHI.

Overall, this line of thinking leads to several methodological possibilities, which may lead to the induction of transient experiences of DP and DR. If frequent, large and variable interoceptive prediction errors can be generated over short periods of time, interoceptive prediction precision may steadily decline. This may lead to a reliance on non-interoceptive sensory modalities (over-integration of sensory data) to reduce interoceptive prediction error, leading to the subsequent generation of DP/DR experiences. Whilst a direct manipulation of interoceptive predictions and errors would be ideal, such as disrupting the afferent feedback signals of one’s heartbeat, such procedures would be physically invasive and difficult to achieve. Alternatively, increasing the dependence on exteroceptive and proprioceptive predictions during a task that involves significant input from interoceptive processes could achieve a similar outcome. For instance, a breathing rate task where individuals are required to focus on a manipulated exteroceptive component (e.g., delayed visual feedback of one’s chest movement), and/or manipulated proprioceptive component (e.g., delayed feedback of the sensation of chest movement) could create a large prediction error in the unaltered interoceptive domain (actual interoceptive sensations arising from breathing). If the task demands require attentional resources be prioritized for exteroceptive and proprioceptive sources of (mis)information, the system’s ability to successfully predict interoceptive signals would be reduced, leading to increasingly imprecise interoceptive predictions and eventually, experiences of DP and DR. As will be discussed, the specific manner in which this methodology could be implemented may also shed light on whether DP and DR experiences could be induced separately.

## Distinguishing Depersonalization and Derealization

Thus far, we have discussed experiences of DP and DR without necessarily considering their differences in the context of experimental findings. Whilst experiences of DP and DR often co-occur, phenomenological accounts of DP and DR suggest they are uniquely different experiences (Colombetti and Ratcliffe, 2012). Further, neuropsychological case studies, in which damage to the right parietal lobe has been associated with DP type experiences, and damage to the occipito-temporal cortex with DR type experiences, suggests they may be due to distinct neuropsychological mechanisms (Sierra et al., 2002b). Additionally, Dewe et al. (2018), found reduced skin conductance responses (which they view as the suppression of autonomic reactions) to simulated threats toward one’s body were correlated with sub-clinical DP traits, whilst reduced skin conductance responses to threats toward others was associated with sub-clinical DR traits. The authors suggest attentional biases toward interoceptive information leads to rapid suppression of autonomic responses in those with DP traits while those with DR traits have an attentional bias toward threatening exteroceptive information which assists them to rapidly suppress autonomic responses to such threats. In cases of mixed DP and DR, they suggest attention to interoceptive information may be so great, that it draws attentional resources away from exteroception, resulting in mixed DP and DR type experiences.

Self-reports of those who experience DR suggest an emotional connection to one’s environment is lost during the experience, whilst connection with the feelings of one’s physical body is lost in those who experience DP (Sierra and David, 2011; Sierra, 2012). From a multi-sensory predictive coding perspective, this may suggest those with DR experience abnormal integration of exteroceptive information with interoceptive processing, resulting in an experience of the world devoid of emotional coloring. In contrast, those with DP may generate abnormally imprecise predictions, generating a loss in one’s subjective sense of bodily feelings and hence the bodily self. In mixed DP/DR experiences, abnormally imprecise interoceptive predictions and disrupted integration of exteroceptive inputs combined with interoceptive processing may be responsible. As suggested by Seth et al. (2011) differences between DDD and certain delusions may be explained by a progression of the same underlying aberrant predictive coding mechanism (e.g., imprecise interoceptive predictions) whereby the Cotard delusion develops in order to reduce prediction error associated with severe DP/DR type experiences. A similar process may explain the differing experiences of DP and DR, where initial stages of dissociation begin with DR, with a progression toward combined DP/DR, followed by DP experiences as the precision of interoceptive predictions decreases and the normal process of integrating exteroceptive information with interoceptive processes becomes disrupted. Whilst Dewe et al. (2018) suggest differences between DP and combined DP/DR may be explained by increased saliency of interoceptive signals, and in DR of exteroceptive signals, we suggest these experiences may actually reflect a progressively increasing bias toward *non*-interoceptive sources of information in an attempt to reduce interoceptive prediction error (by predicting interoceptive processes through attention to non-interoceptive modalities). Initially, individuals may experience DR when the precision of interoceptive predictions is low enough to reduce the salience connected to one’s environment triggering a shift in the relative weighting (gain) of bottom-up exteroceptive versus interoceptive signals in generating high level self-world models but without major disruption to lower level felt-body processing as occurs in DP. As lower level interoceptive prediction precision continues to decline, a transition emerges as one begins to experience a sense of disconnection to the physical body, resulting in a shift in phenomenology from DR to mixed DR/DP and finally to DP. Finally, with growing loss of interoceptive prediction precision comes increasingly flat homeostatic and allostatic regulation of bodily states (active interoceptive inference) and the loss of the sense of connection to one’s physiological self (the felt body) characterizing ‘pure’ episodes of DP.

It may appear counter intuitive to suggest that the phenomenology of DR is replaced by DP as interoceptive precision further unravels with growing homeostatic and allostatic dysregulation. To account for this, we suggest two possibilities, where (1) those with more severe DP are unable to adequality report on their experiences due to a loss of felt connection to themselves, and therefore do not produce reliable self-reported experiences (and may in fact experience DR in addition to DP), (2) as interoceptive prediction precision lowers, a greater dependence on exteroception to generate interoceptive predictions occurs, resulting in further disconnection of interoceptive predictions from updating by interoceptive prediction errors.

**FIGURE 1:**
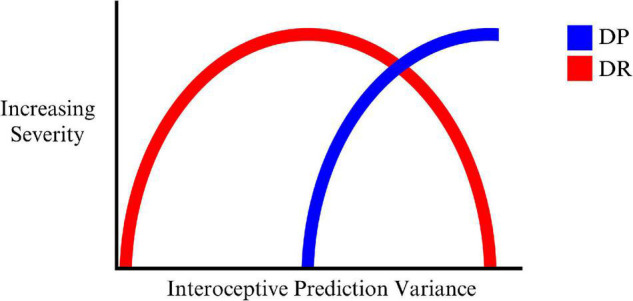
The progression of DR, mixed DP/DR, and DP with increasing variance (decreasing precision) of interoceptive predictions.

On this account DR is the result of an initial attempt to account for growing interoceptive prediction errors by high level model changes corresponding to the sense of reality or connection to the outside world. In this way, one’s sense of feeling strange and unusual is attributed to the external world. This ‘illusion’ can only be maintained at milder levels of imprecision (hence variability) in interoceptive predictions (and corresponding prediction errors), but falls apart as this grows even more imprecise (interoceptive prediction errors becomes too imprecise to be explained solely by the world being unreal), and the organism is unable to model these experiences in relation to the world around them. DP then emerges as a deeper model to account for the consequences of worsening interoceptive precision. This may reflect an ‘inverted U’ type of progression of DR, where DP emerges during the peak of the U as indicated in Figure 1.

Despite these theoretical assumptions, an investigation is required to determine the types of experiences that may emerge when the weighting of a given sensory modality in the generation of interoceptive predictions is adjusted relative to other sensory modalities. More specifically, the potential that DP/DR experiences may emerge when one’s interoceptive predictions are rendered more imprecise relative to predictions in other modalities and more impervious to updating by (correspondingly more imprecise) interoceptive prediction error signals.

Overall, experimental methods capable of inducing experiences of DP and DR in isolation are required to understand the individual psychological mechanisms responsible for these experiences. As such, testing a predictive coding model of DDD capable of distinguishing between DP and DR requires experimental methods that can induce these experiences in isolation. Thus far, current research has failed to adequately achieve this, with experimental DP/DR induction methods to date attempting to induce these experiences without necessarily distinguishing between them. Previous DP/DR induction methods have included auditory/visual stimulation with flashing lights and pulsing sound, stimulus deprivation, staring at a dot on a wall, staring at one’s reflection in a mirror, drug use, hyperventilation, watching a strobe light, and through use of the RHI as previously discussed (Leonard et al., 1999; Mathew et al., 1999; Cohen, 2004; Lickel et al., 2008; Rabellino et al., 2016, 2018; Rosa et al., 2019).

Virtual reality (VR) offers a particularly useful method of inducing experiences of DP and DR, which may be better equipped to manipulate the relative contribution of changes in exteroceptive, proprioceptive, and interoceptive information and map these to changes in the sense of reality and the sense of the felt body than previous researched methods. This stems from its ability to immerse individuals within a completely novel (digital) environment, in which the sensory characteristics of the environment can be altered with a large degree of control and flexibility (Cipresso et al., 2018). For instance, full-body virtual representation may be induced in a VR scenario, where the characteristics of the virtual body could be manipulated in a way unachievable in reality, such as altering the size, structure, morphology, and egocentric visual perspective of the virtual body, and manipulating discrepancies in multi-sensory inputs (Brugger and Lenggenhager, 2014). Whilst most VR technology depends primarily on the manipulation of exteroceptive input, we believe the strength of this technology over non-VR methods that also rely on the manipulation of exteroception relates to its capacity to fine tune the contribution of separate inputs in multi-sensory tasks. For instance, during an implied threat task delivered in a VR environment, the severity of a visual threat (such as falling from a height) can be incrementally increased to a level unsafe within non-VR methods (e.g., increasing the distance of one’s virtual body from the ground before falling). This may result in the generation of interoceptive prediction errors whose magnitude may exceed that which is acceptable in non-VR methods. As an additional example, having individuals direct their attention toward the exteroceptive components of breathing in a virtual body (such as chest movement of one’s virtual body and auditory feedback of breathing) that is delayed relative to one’s actual breathing could reduce the confidence (precision) in interoceptive predictions of cardiac activity. If this delay is increased in a gradual way, a reduction in the precision of interoceptive predictions may occur, potentially leading to experiences of DR, combined DP/DR, and then DP as the delay in exteroceptive input increases relative to interoceptive input, reducing interoceptive prediction precision and increasing the dependence on non-interoceptive modalities to explain away emerging interoceptive prediction error.

This would provide a means of testing whether DP and DR and combined experiences (e.g., DP and DR occurring together) transition from one another as interoceptive prediction precision declines in response to increased sensory input delay (of breathing). However, as the above examples also incorporate a proprioceptive component (e.g., mechanoreceptor activity during chest movement, or vestibular activity detecting falling), a prediction error between exteroception driven predictions and proprioceptive signals would likely be generated during these scenarios in addition to the prediction error generated between interoceptive signals and exteroception based predictions. To control for this, variations on the same experimental conditions could be implemented, but with attention directed toward a different sensory modality in separate conditions. This would hypothetically increase the precision weighting of prediction errors within the targeted sensory modality, reducing the weighting of other modalities in resolving those prediction errors. For instance, directing attention toward the sensation of bodily movement whilst falling in a VR environment may increase the precision of proprioceptive prediction errors, helping resolve these errors without the need for input from other modalities. Indeed, this agrees with Ainley et al. (2016), who suggests attention to non-visual sensory modalities during the RHI reduces the illusory effects by increasing the precision of these modalities, lessening the influence of exteroception in perceiving hand position. Through repetition of these conditions but with varying focuses of attention toward different sensory modalities, the effects of manipulating the precision weighting of a specific modality could be compared to determine the extent to which reducing interoceptive prediction precision influences experiences of DP and DR, relative to manipulating precision in other modalities. This might be manipulated for example by adjusting the tasks utilized during a virtual fall from a height, where individuals could be required to locate specific visual objects within the virtual environment during the fall to engage exteroceptive processes, count breathes whilst falling to engage interoceptive processes, or engage in specific types of head movement (e.g., side to side) to engage proprioceptive processes.

Previously established methods of manipulating multi-sensory integration in a VR environment include altering one’s perspective, such as when individuals view themselves outside of their own body (i.e., out of body experiences); delaying visual feedback to create a sense of asynchronous bodily movement; and by having the virtual body threatened in some way, resulting in alterations in autonomic threat responses within the actual body (Blanke and Metzinger, 2009; Aardema et al., 2010; Kober and Neuper, 2012; Kober et al., 2012; Van Heugten–van der Kloet et al., 2018). These methods often begin by inducing a sense of embodiment in relation to a virtual body, so that one feels as if the virtual body is one’s actual body. However, we are aware of only one study to date (Van Heugten–van der Kloet et al., 2018) which has examined self-reported experiences of both DP and DR as separate experiences during a task manipulating visual perspective.

One major hurdle that may explain this gap may relate to how DP and DR are measured. Generally, self-report measures are used to gauge which dissociative experiences generated during experimental manipulation pertain to DP, and which to DR. As previously indicated, a general distinction between DP and DR pertains to how one loses a sense of connection to one’s body (DP) and one’s environment (DR). In many respects, it is a loss of connection of the relationship between one’s self and body (DP) and one’s self and external environment (DR) which underpins these experiences, where this overall disruption in the self may explain the interconnectedness of these experiences. As these experiences may have elements which phenomenologically overlap (and are often co-occurring), understanding the psychophysiological correlates (and ultimately perhaps markers) of these experiences at both central and peripheral levels, may distinguish the emergence of and relationship between these separate experiences. With VRs potential to actively manipulate multi-sensory perception to induce DP and DR states, a mapping of the neural correlates that emerge during such manipulations, in conjunction with self-report, will help determine if they can be induced in isolation, and what their unique (if unique) neural signatures actually are.

## Heartbeat Evoked Potential, Attention, and Interoception

Evidence regarding the time course and cortical processing pathways of interoceptive signals generated by feedback from cardiac activity can be obtained by using the HEP, which are evoked cortical potentials time locked to cardiac activity (Gentsch et al., 2019). HEPs are commonly observed in EEG data from 200 to 500 ms after the R-peak displayed in the human ECG (Coll et al., 2021). HEP amplitude has been found to correlate with heartbeat detection accuracy (i.e., conscious awareness of heartbeats mediated through attention), and therefore has been quantified as an electrophysiological marker of cardiac interoceptive awareness (Gentsch et al., 2019). Increases in HEP amplitude that occur when directing one’s attention toward one’s heartbeat are believed to reflect increased precision weighting of bottom-up interoceptive signals, where higher HEP amplitudes reflect increased weighting of interoceptive prediction errors (Hohwy, 2012; Petzschner et al., 2019). This allows for the measurement of experimentally induced changes in interoceptive predictive coding within the brain itself. However, Ring and Brener (2018) suggest different methods of heartbeat detection may utilize different interoceptive processes. For instance, counting heartbeats may be reflective of one’s attentional focus, motivation, and predictions held about one’s heart rate, where individuals may simply guess their heart rate based on these predictions rather than detect interoceptive processes and report on these sensations. In contrast, tasks requiring participants to judge whether their hearts are beating simultaneously with exteroceptive stimuli has been suggested to provide a more accurate measurement of one’s ability to perceive cardiac sensations (Ring and Brener, 2018). Despite these criticisms, HEP responses to heartbeat counting may provide a useful measurement of interoceptive processing in DP and DR states as will be discussed.

Thus far, only one study to date has examined heartbeat perception and HEP in individuals with chronic DDD (i.e., combined symptoms of DP and DR; Schulz et al., 2015). Whilst healthy controls showed an increase in HEP amplitude during a heartbeat counting task compared with HEP at rest (e.g., when not engaged in a heartbeat perception task), HEP amplitude did not increase with attention to heart rate for DDD individuals (differences in HEP at rest were not reported; Schulz et al., 2015). Surprisingly, those with DDD initially perform better than controls in at least one form of interoceptive accuracy, heartbeat counting tasks (Michal et al., 2014), but with declining performance occurring over time; contrasting with healthy controls, who showed improved heartbeat counting over time. This suggests individuals with DDD struggle with sustaining attention to interoceptive sensations over time, rather than attending to them *per se* (Salami et al., 2020). However, further examination of this effect is required to determine whether HEP amplitudes become elevated and then decrease over time (reflecting a rapid increase and then gradual decrease in attention to interoceptive signals) in those with DDD during heartbeat counting tasks. Potentially, those with DDD may utilize different brain regions in processing the interoceptive feedback from heartbeats, which may be detected in source localization differences in the time course of the HEP between DDD and non-clinical samples or between VR induced DP and DR conditions and an appropriate baseline condition.

This may occur when other sensory components of one’s heartbeats (e.g., the physical sensation of one’s clothing moving on one’s chest) are given more attention in detecting heartbeats. Thus, dwindling performance on measures of heartbeat detection may reflect an increase in attention to non-interoceptive sources of feedback.

To our knowledge, no other previous studies have examined changes in HEP cortical source activity during heartbeat detection in those with DDD. However, analysis of HEP responses in other psychopathologies suggest HEP responses in individuals with DDD will be unique. For instance, those with obsessive-compulsive disorder displayed elevated HEP responses during heartbeat detection versus rest compared with healthy individuals (Yoris et al., 2017). Further, other studies have shown a similar HEP pattern at rest and during heartbeat detection in depressed participants compared with controls, but with an overall reduction in HEP amplitude. Finally, a higher HEP at baseline has been observed in those with generalized anxiety disorder compared with controls (Terhaar et al., 2012; Yoris et al., 2017; Pang et al., 2019). In addition to the apparent unique HEP amplitude found in those with DP and DR (i.e., no increase in HEP during heartbeat detection), HEP offers a potential insight into the predictive coding mechanisms that may be responsible for this condition. If HEP represents a precision weighted interoceptive prediction error signal as has been proposed by others (Ainley et al., 2016; Petzschner et al., 2019) then attending to one’s heartbeat should increase the precision of these prediction errors, increasingly their likelihood of propagating up the predictive coding hierarchy to update predictions (Banellis and Cruse, 2020). However, if imprecise predictions are responsible for DP and DR, then consistent HEP responses, regardless of one’s focus of attention, could reflect an inability to adjust (or perhaps maintain) the precision weighting of interoceptive predictions versus prediction errors, leading to prediction errors that fail to update predictions. This would result in the maintenance of imprecise interoceptive predictions by failing to adjust predictions in the face of prediction error. Given the reported loss in sense of self experience by those with DP, as well as alterations in perception of the environment in relation to one’s self as experienced in DR, a deeper understanding of HEP responses in those with DP and DR is highly desirable.

## Conclusion and Future Directions

Based on the preceding discussion on what HEP data reflects and its relevance to the phenomenal experiences of DP and DR, future studies could utilize paradigms in which HEP data is obtained during manipulation of expectancy errors between exteroceptive, proprioceptive and interoceptive dimensions of sensory information generated in VR in order to study neural mechanisms underlying DP and DR. In particular in conditions where a single sensory domain drives changes in the relative precision of predictions within the interoceptive domain. Whilst HEP responses in those with persistent DDD have previously been discussed, little is known about HEP responses in those subjected to transient experiences of DP and DR. Extending on previous literature, we hypothesize that an increase in HEP would likely occur in a non-clinical sample of individuals when directing attention toward one’s heart rate, whereas this change is likely to be small (or non-existent) in non-clinical cases experiencing a transient DP/DR experience, similar to HEP responses in those with DDD [as in research conducted by Schulz et al. (2015)].

If cortical sources engaged by processing in the HEP can be used as an indicator of phenomenological aspects of DDD, inducing DP/DR experiences in psychologically normal individuals (i.e., without otherwise self-reported DDD symptoms) could produce similar experiences and HEP patterns as seen in those with DDD. However, if experiences of DP and DR are simply different points along the same continuum of interoceptive prediction imprecision, then this should be reflected in the HEP amplitude, with lower relative to higher amplitudes reflecting DR, combined DP/DR, and DP states, respectively, during a heartbeat detection task. As such, examining the effect of DP and DR in relation to brain regions engaged in processing HEPs is indicated in order to determine the HEPs role as an indicator of interoceptive predictive processing in both DP and DR.

As previously indicated, an example of a task that could achieve the above aims may include a manipulated breathing task, in which the exteroceptive feedback is delayed relative to interoceptive feedback whilst individuals are tasked with attending to either exteroceptive, interoceptive, or proprioceptive sources of information. The subsequent effect on HEP during a heartbeat detection task and subjective feelings of DP and DR can then be compared in order to determine if these experiences/states can be successfully induced within a VR environment, and be identified by HEP amplitude and underlying cortical sources, particularly when attention is directed toward non-interoceptive sources of information during this task. More specifically, if lack of HEP processing change during a heartbeat detection task is an indicator of experiences of DP and/or DR, the strength of DP or DR induction (in non-clinical individuals) may be inversely associated with HEP amplitude increase during a heartbeat detection task completed immediately following DP/DR induction, with HEP amplitude during the heartbeat detection task becoming progressively closer to HEP at rest as DP/DR induction strength increases (and as the phenomenological experience transitions from DR, mixed DP/DR, to ‘pure’ DP) and with those brain regions interacting with the interoceptive signals generating the HEP. If successful, this paradigm would provide further clarification as to whether:

a) Experiences of DP and DR can be induced separately, and their relationship to each other,
b) HEP changes can be used as a reliable neural correlate of DP and DR based on self-reported symptoms, with HEP amplitudes during heartbeat detection becoming closer to baseline as DR transitions to mixed DP/DR, and then DP.
c) HEP amplitudes and/or underlying cortical sources, in those with DP and DR, change over time and with attention toward/away from interoceptive signals,
d) DP and/or DR can be manipulated individually by altering the relative precision of interoceptive predictions driven by exteroceptive, proprioceptive and interoceptive sensory modalities, providing further evidence for the role of interoceptive predictive coding mechanisms within the human mind-body nexus.

The methods proposed here to induce transient experiences of DP and DR in healthy volunteers are all based upon leveraging the capabilities of VR technology to create mismatches and between processing in exteroceptive, proprioceptive and interoceptive sensory streams and hence the cross modal predictions originating from generative models in one stream on top-down predictions (and corresponding prediction errors) in another stream as well as integration of perceptual generative models in higher order models of the self and self-world relationship. As such they are inherently informed by the predictive processing framework and offer the opportunity to fine tune interventions targeted at manipulating parameters inherent to such models such as prediction updating, prediction error, precision and confidence between different hierarchical levels and perceptual processing streams in order to test different models of functioning in such processing hierarchies in states of DR and DP. The approach of the current authors is to focus on interoceptive predictive coding related to the experience of feeling states of the body (homeostatic and allostatic salience) and emotions (the salience of changing self-world possibilities; Craig, 2009) which are greatly diminished, if not ablated, in the core phenomenology of DDD. Indeed, it is these aspects of DDD experience which are the primary source of distress for sufferers of the disorder and form the target of clinical interventions.

Consequently our initial application of these methods is directed at interactions in the predictive processing hierarchy of interoceptive inputs running from posterior to mid to anterior insula (Craig, 2009; Barrett and Simmons, 2015) as well as the efferent and afferent connections of each level in this processing hierarchy with other perceptual processing hierarchies, cognitive and attentional processes and brain regions modulating autonomic nervous system activity (active interoceptive inference). As such our focus is on factors which may reduce the gain of interoceptive prediction errors and the updating of predictive models in mid (bodily feelings hence DP related) and anterior (emotion or self-world and hence DR related) insula. Such factors may be targets for VR based therapeutic interventions in individuals with DDD. However, predictive processing is a developing framework (e.g., Sandved-Smith et al., 2021) rather than a theory, and alternative models are possible (indeed desirable) within this framework (Smith et al., 2019).

Ciaunica et al. (2021) developed a model which focuses on giving an account of DDD alterations in the sense of self, in particular the experience of the self as mechanical and robotic, as an object which is observed from the outside rather than lived from the inside. Their model is based upon a failure to attenuate sensory signals due to self-initiated actions, leading to a pervasive sense of alienation or non-selfhood in relation to these sensations. In conjunction with this they propose that a lack of confidence in predictions of the sensory consequences of self-generated actions drives enhanced attention to metacognitive processes leading to further opacity of normally transparent high level self-regulation and hence the experience of the self as an external object. It may be inferred that Ciaunica et al. (2021) and ourselves expect diametrically opposite effects in terms of prediction errors and prediction updating, although we focus specifically upon interoceptive processing and their model does not emphasize particular sensory modalities. Methods of the type we propose, in conjunction with co-recorded EEG (allowing for source localization) and ECG (allowing for HEP analysis) are well suited to testing the different predictions of such diverse predictive processing models. Fundamentally knowledge of the functional neural underpinnings of DP and DR can only grow when computational models are brought into contact with relevant neural data. Such knowledge is essential for the development and testing of effective interventions for those suffering DDD and dissociation associated with other distressing psychological conditions.

## Data Availability Statement

The original contributions presented in the study are included in the article/supplementary material, further inquiries can be directed to the corresponding author.

## Ethics Statement

Ethical review and approval was not required for the study on human participants in accordance with the local legislation and institutional requirements. Written informed consent for participation was not required for this study in accordance with the national legislation and the institutional requirements.

## Author Contributions

AG wrote the manuscript under the supervision of GJ and BS. All authors were equally involved in the development of the hypothesis and theory presented in this review.

## Conflict of Interest

The authors declare that the research was conducted in the absence of any commercial or financial relationships that could be construed as a potential conflict of interest.

## Publisher’s Note

All claims expressed in this article are solely those of the authors and do not necessarily represent those of their affiliated organizations, or those of the publisher, the editors and the reviewers. Any product that may be evaluated in this article, or claim that may be made by its manufacturer, is not guaranteed or endorsed by the publisher.
